# Composition of the Essential Oil and Insecticidal Activity of *Launaea taraxacifolia* (Willd.) Amin ex C. Jeffrey Growing in Nigeria

**DOI:** 10.3390/foods9070914

**Published:** 2020-07-11

**Authors:** Moses S. Owolabi, Akintayo L. Ogundajo, Azeezat O. Alafia, Kafayat O. Ajelara, William N. Setzer

**Affiliations:** 1 Department of Chemistry, Lagos State University, P.M.B. 001, LASU, Lagos 102001, Nigeria; ogundajotayo@yahoo.com; 2 Department of Zoology and Environmental Biology, Lagos State University, P.M.B. 001, LASU, Lagos 102001, Nigeria; honeylafia@yahoo.com (A.O.A.); kajelara@yahoo.com (K.O.A.); 3 Department of Chemistry, University of Alabama in Huntsville, Huntsville, AL 35899, USA; 4 Aromatic Plant Research Center, 230 N 1200 E, Suite 100, Lehi, UT 84043, USA

**Keywords:** essential oil composition, limonene, sabinene, citronellal, *Sitophilus oryzae*

## Abstract

The rice weevil (*Sitophilus oryzae*) is a pest of stored grain products such as rice, wheat, and corn. Essential oils represent a green environmentally-friendly alternative to synthetic pesticides for controlling stored-product insect pests. *Launaea taraxacifolia* is a leafy vegetable plant found in several parts of Nigeria. The leaves are eaten either fresh as a salad or cooked as a sauce. The essential oil obtained from fresh leaves of *L. taraxacifolia* was obtained by hydrodistillation and analyzed by gas chromatography/mass spectrometry (GC-MS). Twenty-nine compounds were identified, accounting for 100% of the oil composition. The major component classes were monoterpene hydrocarbons (78.1%), followed by oxygenated monoterpenoids (16.2%), sesquiterpene hydrocarbons (2.1%), oxygenated sesquiterpenoids (0.3%), and non-terpenoid derivatives (3.3%). The leaf essential oil was dominated by monoterpene hydrocarbons including limonene (48.8%), sabinene (18.8%), and (*E*)-β-ocimene (4.6%), along with the monoterpenoid aldehyde citronellal (11.0%). The contact insecticidal activity of *L. taraxacifolia* essential oil against *Sitophilus oryzae* was carried out; median lethal concentration (LC_50_) values of topical exposure of *L. taraxacifolia* essential oil were assessed over a 120-h period. The LC_50_ values ranged from 54.38 μL/mL (24 h) to 10.10 µL/mL (120 h). The insecticidal activity of the *L. taraxacifolia* essential oil can be attributed to major components limonene (48.8%), sabinene (18.8%), and citronellal (11.0%), as well as potential synergistic action of the essential oil components. This result showed *L. taraxacifolia* essential oil may be considered as a useful alternative to synthetic insecticides.

## 1. Introduction

Insects such as *Callosobruchus maculatus* (Fabr.) (bruchid beetle), *Sitophilus granarius* (L.) (wheat weevil), *S. oryzae* (L.) (rice weevil), *S. zeamais* (Motsch.) (maize weevil), and *Tribolium castaneum* (Herbst) (red flour beetle), are important pests that attack stored grains, causing widespread economic losses [1,2,3]. The long-term use of synthetic insecticides to control these pests has become problematic, however. Compounds such as chlorinated hydrocarbons, organophosphates, carbamates, etc., tend to be toxic to non-target organisms such as mammals, birds, and fish [4,5,6], they are persistent in the environment [7,8,9,10], and many stored-grain insect pests have developed insecticide resistance [11,12,13]. Essential oils have emerged as viable alternatives to synthetic pesticides for control of stored-grain insect pests; they are generally non-toxic to mammals, birds, fish, or humans, have limited persistence, are readily biodegradable, and are renewable resources [14,15,16,17].

*Launaea taraxacifolia* (Willd.) Amin ex. C. Jeffrey (syn. *Lactuca taraxacifolia* (Willd.) Schumach, wild lettuce) is a leafy vegetable plant belonging to the Asteraceae (Compositae). The family consists of roughly 1100 genera, and 20,000 species distributed across several countries including Mexico, West Indies, Central and South America, Europe, North Africa, and tropical West African countries like Ghana, Senegal, Benin, and Nigeria [18]. *L. taraxacifolia* is a wild erect perennial herb that grows up to 1–3 m in height with 3−5 pinnately lobed leaves at the base of the stem in a rosette form. The plant is found singly or in clusters of rocky soil, but it is also cultivated in small open gardens near homes for family consumption. The leaves are eaten fresh as a salad or cooked as sauces [18,19,20,21,22,23,24]. The plant is known as ‘efo yanrin’ among the Yorubas of the southwestern part of Nigeria, ‘ugu’ among the Ibos of the eastern part of Nigeria, and ‘nonon barya’ among the Hausas of the northern part of Nigeria. Minerals, proteins, flavonoids, fatty acids, and vitamins have been reported to be found in the leaves of *L. taraxacifolia* [25,26]. The nutritional aspects of *L. taraxacifolia* have been reviewed [27,28]. The antioxidant and antiviral activities as well as the use of *L. taraxacifolia* leaves in treatment and control of blood cholesterol levels, blood pressure, and diabetes have been reported [29,30]. Phytochemical studies of *L. taraxacifolia* revealed that the plant possesses chemical classes such as phenolic glycosides, flavonoids, saponins and triterpenoids, which are known to have phytotherapeutic value for humans [25,31,32,33,34]. To the best of our knowledge, there is little or no information on the composition of the essential oil or the insecticidal activity of *L. taraxacifolia*. Therefore, the present research was undertaken with the aim of investigating the essential oil composition and evaluating the insecticidal potential of *L. taraxacifolia* leaves from southwestern Nigeria.

## 2. Materials and Methods

### 2.1. Plant Materials

The leaves of *L. taraxacifolia* were collected from Ipara, Badagry (6°4′54.07″ N and 2°52′52.75″ E) Lagos state, Nigeria. Botanical identification was done at the Herbarium, University of Lagos, Nigeria, where a voucher specimen (LUH: 7959) was deposited. Fresh leaves of *L. taraxacifolia* were cut into pieces, air dried, and pulverized in a blender to increase the surface area. A 450-g sample of blended *L. taraxacifolia* was hydrodistilled for 4 h in an all-glass modified Clevenger-type apparatus according to British Pharmacopoeia [35]. The obtained essential oil was stored in a sealed glass bottle with a screw lid cover under refrigeration at 4 °C until ready for use. Oil yield was calculated on a dry weight basis.

### 2.2. Gas Chromatographic–Mass Spectral Analysis

The chemical composition of *L. taraxacifolia* essential oil was determined by gas chromatography–mass spectrometry (GC-MS) using a Shimadzu GCMS-QP2010 Ultra operated in the electron impact (EI) mode (electron energy = 70 eV), scan range = 40–400 atomic mass units, with a scan rate of 3.0 scans per s, with GC-MS solution software. The GC column was a ZB-5 fused silica capillary column (30 m length × 0.25 mm inner diameter) with a 5% phenyl-polymethylsiloxane stationary phase and a film thickness of 0.25 μm. Helium gas was used as a carrier gas with column head pressure of 552 kPa at a flow rate of 1.37 mL/min. The injector temperature was 250 °C and the ion source temperature was 200 °C. The oven temperature of 50 °C was initially programmed for the GC and gradually increased at 2 °C/min to 260 °C. The sample (5% w/v) was dissolved in dichloromethane and 0.1 μL of the solution was injected using a split injection technique (30:1). Identification of the essential oil components was achieved by comparing the retention indices determined with respect to a homologous series of *n*-alkanes, and by comparison of the mass spectral fragmentation patterns with those stored in the MS databases [36,37,38,39].

### 2.3. Insecticidal Activity Screening

The essential oil was screened for insecticidal activity based on the method of Ilboudo and co-workers [40] with modifications. *Sitophilus oryzae* (L.) (rice weevil) were reared on whole rice (10:1 w/w). Adult insects, 1–7 days old, were used for contact toxicity tests. The insects were cultured in a dark growth chamber at a temperature of 27 ± 1 °C with relative humidity of 65 ± 5%. The insecticidal activity of *L. taraxacifolia* oil against *S. oryzae* (rice weevil) was evaluated by treatment of Whatman No. 1 filter paper discs with the essential oil diluted in ethanol. The required quantities of oil (0.10, 0.20, 0.30, and 0.40 µL) were diluted to 1 mL with ethanol and applied to filter paper discs, respectively. Permethrin (0.6% w/w) and ethanol were used as positive and negative controls, respectively. The solvent was allowed to evaporate from the filter paper, which was then placed into polyethylene cups (80 mm diameter). Ten well-fed mixed sex adult *S. oryzae* were introduced into the polyethylene cups, containing 20 g uninfected rice grains, and covered with a muslin cloth, held in place with rubber bands. Each treatment was replicated four times. Control experiments were set up as described as above without the essential oil. The experiment was arranged in a complete randomized design on a laboratory bench. The insect was considered dead when the legs or antennae were observed to be immobile. Insect mortalities were investigated by observing the recovery of immobilized insects after 24 h intervals for 120 h and the percentage of insect mortality was corrected using the Abbott formula [41]. Probit analysis [42] using XLSTAT version 2018.1.1.60987 (Addinsoft™, Paris, France) was used to estimate median lethal concentration (LC_50_) values and insect toxicity data were analyzed using one-way ANOVA Tukey’s honestly significant difference test.

## 3. Results and Discussion

### 3.1. Essential Oil Composition

The essential oil from *L. taraxacifolia* was obtained by hydrodistillation with a yield of 1.68% as a pale-yellow essential oil, which was analyzed by GC-MS. The chemical composition of the leaf volatile oil of *L. taraxacifolia* is listed in Table 1. A total of 29 compounds were identified, accounting for 100% of the essential oil composition. The major chemical classes were monoterpene hydrocarbons (78%) and oxygenated monoterpenoids (16.2%), followed by sesquiterpene hydrocarbons (2.1%), oxygenated sesquiterpenoids (0.3%), and non-terpenoid derivatives (3.3%). The leaf essential oil was dominated by monoterpene hydrocarbons including limonene (48.8%), sabinene (18.8%), and (*E*)-β-ocimene (4.6%), along with the monoterpenoid aldehyde citronellal (11.0%). The chemical constituents of *L. taraxaciflora* essential oil have not been previously reported to the best of our knowledge. However, a phytochemical study and antioxidant and bacterial screening of the leaf extract of *L. taraxacifolia* have been reported [43].

### 3.2. Insecticidal Activity

The contact toxicity of *L. taraxacifolia* against *S. oryzae* revealed considerable differences in insect mortality rate to the essential oil with different concentrations and different exposure times. Table 2 shows that at a dose of 10.00 μL/mL, the volatile oil produced 25.00% mortality after 48 h (not significantly different than the negative EtOH control) and 52.50% after 120 h (significantly higher toxicity than the EtOH control). The essential oil produced 30.00%, 47.50%, 60.00%, and 75.00% mortality after 48, 72, 96, and 120 h at a dose of 20.00 μL/mL, respectively, while a dose of 30.00 μL/mL yielded a mortality rate of 42.50%, 57.50%, 75.00%, and 75.00%, respectively, over the same period of time. With longer contact times (≥48 h), 20 μL/mL and 30 μL/mL concentrations of *L. taraxacifolia* essential oil was significantly more toxic than the EtOH control, but less toxic than the permethrin positive control. The highest concentration of 40.00 μL/mL produced a mortality of 97.50%, and 100.00% after 96 and 120 h, respectively, which is significantly comparable to the permethrin positive control. Permethrin (0.6% w/w) against *S. oryzae* caused 40.0% mortality with 24 h of exposure and 100.0% mortality after 48 h. The negative control showed no appreciable activity against *S. oryzae* until after 120 h.

Median lethal concentration (LC_50_) values at 95% confidence limits over exposure of *L. taraxacifolia* essential oil were assessed and are shown in Table 3. After 120 h of exposure with an increase in concentration at regular intervals of 24 h, the LC_50_ values were 54.38, 31.64, 21.48, 16.38, and 10.10 µL/mL, respectively. In this study, the essential oil of *L. taraxacifolia* demonstrated contact toxicity to *S. oryzae*, since it had higher insecticidal activity with increasing essential oil concentration and exposure time. This result showed *L. taraxacifolia* essential oil to have promising insecticidal activity against *S. oryzae* and therefore may be considered as a useful, environmentally benign alternative to synthetic insecticides.

To best of our knowledge, there have been no previous literature reports on the insecticidal activity of *L. taraxacifolia* essential oil against *S. oryzae* insect pest. However, contact toxicity of both limonene and sabinene, the major chemical components in this present study, have shown insecticidal activity against *S. oryzae* [44]. Limonene has been previously reported to have a moderate contact effect against *S. zeamais* (LD_50_ values of 198.66 μg/cm^2^) and *S. oryzae* (with LD_50_ of 260.18 μg/cm^2^) [45] as well as fumigant toxicity against *S. oryzae* (24-h LC_50_ 61.5 μL/L) [46]. Garcia et al. reported that limonene showed contact toxicity against *T. castaneum* [47]. Sabinene, on the other hand, demonstrated weaker insecticidal activity against *S. oryzae* (24-h LC_50_ 463 μL/L) [44]. Interestingly, the *S. oryzae* fumigant insecticidal activities of limonene and sabinene parallel the acetylcholinesterase (AChE) inhibitory activities; AChE IC_50_ = 9.57 μL/mL and 85.03 μL/mL, respectively, for limonene and sabinene [48]. Furthermore, the binary combination of limonene + sabinene showed synergistic AChE inhibition [48]. The insecticidal activity of the *L. taraxacifolia* essential oil could be attributed to those known major components and the resulting synergistic action of the monoterpene hydrocarbons limonene (48.8%) and sabinene (18.8%).

The major aldehyde essential oil component, citronellal (11.0%), has also shown contact insecticidal activity against *Musca domestica* [49] and *S. oryzae* [50] and fumigant insecticidal activity against *T. castaneum* [51] and *S. zeamais* [52]. (–)-Citronellal has also shown AChE inhibitory activity with IC_50_ of 18.4 mM [50]. The contact toxicities of bornyl acetate, (+)-limonene, myrcene, α-phellandrene, α-pinene, sabinene, and terpinolene, essential oil constituents obtained from leaves of *Chamaecyparis obtusa*, against *Callosobruchus chinensis* (L.) and *Sitophilus oryzae* (L.) have been reported [44]. The insecticidal activity of the essential oil components 1,8-cineole, *p*-cymene, α-pinene, and limonene has been previously reported with the order of activity 1,8-cineole > *p*-cymene > α-pinene > limonene [46]. Abdelgaleil et al. reported a comparative study of eleven monoterpenes contact and fumigant toxicity: camphene, (+)-camphor, (−)-carvone, 1-8-cineole, cuminaldehyde, (L)-fenchone, geraniol, (−)-limonene, (−)-linalool, (−)-menthol, and myrcene, against two important stored products insects, *S. oryzae,* and *T. castaneum*, and discovered that the toxicity varied according to insect pest with *S. oryzae* more susceptible to most of the components than *T. castaneum* [53].

## 4. Conclusions

This study investigated the essential oil composition and evaluated the insecticidal potential of *L. taraxacifolia* leaves for the first time as a potential substitute to synthetic insecticides. *L. taraxacifolia* offers an advantage in Nigeria due to its accessibility and renewability. Despite many advantages of medicinal plants, especially the essential oils, further studies need to be conducted to ascertain the safety of this essential oil before its practical use as an insecticide for controlling stored product insect pests. In addition, while the insecticidal properties of *L. taraxacifolia* essential oil are promising, this work is preliminary and future investigations extrapolating the use of the essential oil under grain-storage conditions should be pursued. In addition, studies on the controlled-release formulations of the essential oil could be examined to curb some of the challenges of essential oil treatments such as rapid degradation, volatility, and low bioavailability of the essential oils.

## Figures and Tables

**Table 1:** The chemical constituents of *Launaea taraxacifolia* leaf essential oil.

Constituents	RI_calc_^1^	RI_db_^2^	Relative Abundance (%)
α-Pinene	941	933 [37]	0.9
Sabinene	976	971 [37]	18.8
Myrcene	993	991 [37]	2.2
α-Terpinene	1018	1018 [37]	0.6
Limonene	1032	1030 [37]	48.8
(*Z*)-β-ocimene	1042	1034 [37]	0.9
(*E*)-β-ocimene	1052	1045 [37]	4.6
γ-Terpinene	1062	1058 [37]	1.0
Terpinolene	1088	1086 [36]	0.4
Linalool	1101	1099 [38]	3.1
Citronellal	1155	1151 [38]	11.0
Terpinen-4-ol	1178	1180 [37]	1.4
1-Dodecene	1192	1192 [39]	0.5
*n*-Dodecane	1200	1200 [36]	0.5
Neryl acetate	1366	1366 [39]	0.7
1-Tetradecene	1392	1388 [36]	0.5
*n*-Tetradecane	1400	1400 [36]	0.2
β-Caryophyllene	1420	1417 [36]	1.5
α-Humulene	1456	1452 [36]	0.1
Bicyclogermacrene	1495	1497 [38]	0.3
Germacrene B	1556	1559 [36]	0.2
Caryophyllene oxide	1581	1582 [36]	0.3
1-Hexadecene	1592	1588 [36]	0.7
Pentadecanal	1712	1715 [38]	1.0
Monoterpene hydrocarbons			78.1
Oxygenated monoterpenoids			16.2
Sesquiterpene hydrocarbons			2.1
Oxygenated sesquiterpenoids			0.3
Non-terpene derivatives			3.3
Total identified (%)			100

^1^ RI_calc_ = Kovats retention index determined with respect to a homologous series of *n*-alkanes on a ZB-5 column. ^2^ RI_db_ = Retention index from the databases [36,37,38,39].

**Table 2:** Contact insecticidal effects of *Launaea taraxacifolia* essential oil on adult mortality of *Sitophilus oryzae* reared on rice grains 120 h after treatment.

Mean % Mortality (±SE) ^1^					
Concentration (µL/mL)	24 h	48 h	72 h	96 h	120 h
10.00	7.50 ± 5.00 ^cd^	25.00 ± 12.91 ^cd^	25.00 ± 12.91 ^de^	25.00 ± 12.91 ^c^	52.50 ± 17.08 ^c^
20.00	15.00 ± 5.77 ^cd^	30.00 ± 14.14 ^c^	47.50 ± 17.08 ^cd^	60.00 ± 14.14 ^b^	75.00 ± 5.77 ^b^
30.00	22.50 ± 9.57 ^bc^	42.50 ± 12.58 ^bc^	57.50 ± 9.57 ^bc^	75.00 ± 5.77 ^b^	75.00 ± 5.77 ^b^
40.00	45.00 ± 17.32 ^a^	65.00 ± 12.91 ^b^	75.00 ± 5.77 ^b^	97.50 ± 5.00 ^a^	100.00 ± 0.00 ^a^
EtOH control	2.50 ± 5.00 ^d^	5.00 ± 5.77 ^d^	10.00 ± 8.16 ^e^	12.50 ± 9.57 ^c^	25.00 ± 5.77 ^d^
Permethrin	40.00 ± 0.00 ^ab^	100.00 ± 0.00 ^a^	100.00 ± 0.00 ^a^	100.00 ± 0.00 ^a^	100.00 ± 0.00 ^a^
F-value, DF ^2^	15.08, 5	37.44, 5	39.69, 5	62.21, 5	51.13, 5

^1^ Mean followed by different letters in a column is significantly different at (*p <* 0.05). Insect toxicity data were analyzed using one-way ANOVA followed by Tukey’s test. ^2^ Degrees of freedom.

**Table 3:** Median lethal concentrations (LC_50_, μL/mL, and 95% confidence limits) of *Launaea taraxacifolia* essential oil against *Sitophilus oryzae*.

	Contact Time				
	24 h	48 h	72 h	96 h	120 h
LC_50_ (95% confidence limits)	54.38 (39.26−133.8)	31.64 (23.86−55.67)	21.48 (16.62−27.21)	16.38 (13.56−18.78)	10.10 (5.67−13.31)
